# The Gas Exhibition. II.—Institutional Applications Demonstrated

**Published:** 1913-10-18

**Authors:** 


### THE GAS EXHIBITION.

### II. Institutional Applications Demonstrated.

In passing on through a large number of exhibits of great general interest, two minor points will strike the institutional visitor. One of these is the attempt to emulate one of the conveniences of electric lighting by means of an apparatus which will light the gas lamp from a distance. The prices of these will probably be sufficient for hospital officers—8s. 6d. each for the first type and 17s. 6d. each for the second type. Another accessory whose usefulness is vitiated by its price is a small meter which can be fixed by the side of an individual gas fire. This is specially recommended by the manufacturers for hotels where a client may, by its means, be charged merely with the amount of gas he has consumed. Obviously this would be equally useful in hospitals as a check on extravagant use, especially where gas fires are fixed in the residents' quarters. The price of each meter, however, is 16s. 8d., and it cannot be had on hire.

Gas fires constitute, of course, a special feature of the Exhibition. They are numerous and of every possible kind. They have been thoroughly developed not only from the heating but also from the æsthetic standpoint, and the visitor will be struck by their adaptability to every possible situation. In all the latest types the asbestos is in perpendicular straight lines, instead of being, as formerly, in loose pieces (except in some of the larger grates, where small pieces of black asbestos give the appearance of a glowing coke fire). A valuable feature of some of the gas fires is a cock which permits a definite portion of the fire to be shut off, this being much more satisfactory than the usual practice of lowering all the jets simultaneously.

Amongst the gas fires in Section L will be found a small radiator of a new type, known as the "Vitreosil" radiator. This consists of three parallel tubes about 12 inches high and 2 inches in diameter, made of quartzite, a material which can be heated up to 2,000° F. Inside each of these tubes is a screw-shaped pipe, also made of quartzite, through which the gas passes. The radiator throws out quite a good heat and burns only 15 cubic feet per hour.

Near by (Section M) is an apparatus for bringing in air, cleansing it, and passing it over one or more radiators to impart heat to it. This apparatus is made by Messrs. George Wilson, of Coventry.

Close to this is the hospital ward. The space is very limited, and there is no actual ventilation, so that it is difficult to judge properly the effect of the gas lights. The main illumination is by a three-light indirect bowl pendant, giving a soft light of 200 candle-power. A bedside lamp is fixed, adaptable to suit the convenience of the patient. A doctor's bracket-lamp is also attached to the wall, with a Telschow fitting. This is specially suitable for throat work, the gas light being preferred for this by many surgeons. The apparatus appears, however, too cumbersome and too costly (three guineas) to be employed anywhere except in a special department. Gas lights on metallic tubing are not shown, though one would have thought that these would have provided the nearest approach to the electric handlamp. The heating of the ward is done by a gas fire in combination with radiators fed with gas-heated water. The size and number of such fires will, of course, depend in actual practice on the size of the ward to be heated; in this case, the fire shown is estimated to warm an area of 18 feet square at a cost of about 1¼d. per hour. Other fittings of the ward include a water steriliser by C. Lumley and Co., which is rather a large and clumsy-looking affair; a small instrument steriliser, heated by a gas tube; and a small cooker fitted with the "Larkin" boiler already referred to. Before leaving the ward, it may be apposite to remark that the gas company (generally speaking) will undertake the maintenance of mantles at 2s. per burner per annum, and they reasonably ask that this should be compared with the cost of renewing electric lamps, which is rarely less than 4s. per point per annum.

Section 132 is a nurses' sitting-room, nicely lighted with two wall-brackets and a pendant, and heated with a gas fire. The effect in the comparatively small space is one of warmth and comfort.

In an adjoining lobby are a gas refuse destructor and a gas hot-water boiler. The destructor is a "Flavelle," made by the Imperial Stove Co., and it is said to be in use at the Hampstead General Hospital. The 3 feet 6 inches by 1 foot 8 inches destructor is capable of consuming 4½ cwt. of refuse in ten hours, and its price is £6. The hot-water boiler is by T. Potterton, and is in triplicate. The whole apparatus shown would cost about £45, and it is capable of providing six baths an hour at 100° F., each of 20 gallons. Although not primarily intended for a radiator system, there is no doubt the apparatus could be successfully adapted for that purpose in the case of a small institution.

Baths of various kinds are shown, supplied with water heated by Ewart's geysers. These appear to be very satisfactory, though they obviously could not be used with economy in a hospital in place of the circulating hot-water system. At the same time, those of a small type would be very useful in a ward kitchen or anywhere where boiling water must be had at any moment day or night, or where a partial failure of the hot-water system is likely to have serious results. The gas consumption of these small geysers is not very great, 25 gallons of water at 100° being produced for about 1d.

To the general question of the hygienic value of gas for lighting and heating the authorities of the Exhibition have paid special attention, and the Inquiry Bureau will supply a great deal of literature dealing with this point. It is noteworthy that scientists of established reputation, such as Professor Vivian Lewes and Dr. Rideal, have pronounced unequivocally in favour of gas from the hygienic aspect. Professor Lewes some time ago made some experiments in a room having a capacity of 2,700 cubic feet, and obtained the result that when the room was lighted by two Welsbach "C" burners and mantles as a pendant, each consuming 4 cubic feet of gas per hour, the air at the breathing level in the room was far purer and fresher than when three 16 candle-power electric lamps were used, and that the result became the more markedly in favour of gas the larger the number of people in the room. These conclusions he confirmed by later experiments; while as recently as last October the same scientist stated in a public lecture that with a modern gas-stove one may heat a room under conditions every bit as hygienic as with a coal fire, while gas produces a movement of the air very valuable as an aid to ventilation. Such assertions, when corroborated by scientific arguments and researches, cannot but carry great weight.

We need only add of the Gas Exhibition that it is arranged in a very attractive and thoughtful manner. The courtesy and helpfulness of the officers of the Exhibition in no case degenerate to importunity. The object of the organisers has been to stimulate interest in gas and its applications, and to avoid altogether the appearance of advertising any special firm or appliance.

